# Unusual extension of a left-sided retroperitoneal lipoma into the thigh: A case report

**DOI:** 10.1097/MD.0000000000043528

**Published:** 2025-07-25

**Authors:** Abdul Muqeet, Emad Uddin Sajid, Mohammad Faisal Ibrahim, Anfal Atif, Absar Hyder, Muhammad Imran Siraj, Nour Fakih

**Affiliations:** a Department of Surgery, Dow University of Health Sciences, Karachi, Pakistan; b Department of Medicine, Dow University of Health Sciences, Karachi, Pakistan; c Department of Natural Sciences, Lebanese American University, Beirut, Lebanon.

**Keywords:** case report, retroperitoneal liposarcoma, soft tissue sarcoma, surgical resection, thigh extension

## Abstract

**Rationale:**

Retroperitoneal liposarcomas are rare mesenchymal tumors often diagnosed late due to asymptomatic growth. While they typically extend into adjacent retroperitoneal structures, invasion into the thigh is exceedingly rare, posing unique diagnostic and therapeutic challenges.

**Patient concerns:**

A 45-year-old South Asian female presented with a progressively enlarging swelling in the left upper thigh, extending into the left inguinal and flank regions, accompanied by intermittent pain.

**Diagnoses:**

Imaging revealed a large, lobulated, fat-attenuating mass measuring 11 × 9 cm in the axial plane with a craniocaudal extent of 30.7 cm, spanning from the retroperitoneum to the thigh. Histopathology confirmed a well-differentiated liposarcoma.

**Interventions:**

The patient underwent surgical resection via a combined laparotomy and thigh incision, achieving complete excision with negative margins.

**Outcomes:**

Postoperative recovery was uneventful. At 6-month follow-up, there was no tumor recurrence or functional limitation reported.

**Lessons:**

This case highlights the importance of comprehensive imaging and meticulous surgical planning in managing atypical tumor extensions such as retroperitoneal liposarcomas invasion into the thigh. Given the rarity of this presentation, it contributes valuable insights into clinical and surgical management strategies for similar cases.

## 1. Introduction

Retroperitoneal liposarcomas (RPLs) are rare mesenchymal tumors, accounting for 13% to15% of all soft tissue sarcomas.^[1]^ The estimated annual incidence is 1 to 2.5 cases per million individuals ^[2,3]^, with these malignancies typically originating in the retroperitoneal space. Due to the expansive capacity of this region, RPLs often remain asymptomatic until they reach a substantial size, leading to compression of adjacent structures and symptoms such as abdominal discomfort or a palpable mass.^[4,5]^ Late-stage diagnoses are common, complicating treatment and negatively impacting prognosis.^[1,6]^ Studies suggest that symptom presence at diagnosis, including tumor palpability and abdominal pain, is an independent prognostic factor, correlating with higher recurrence rates and poorer outcomes.^[7]^

Histologically, RPLs are classified into well-differentiated, dedifferentiated, myxoid, and pleomorphic subtypes, with well-differentiated tumors generally associated with better prognoses.^[6]^ Complete surgical resection with negative margins remains the cornerstone of treatment, often requiring multi-organ resection for curative outcomes.^[3]^ However, recurrence rates remain high, and adjuvant therapies, including chemotherapy and radiotherapy, have limited efficacy due to the tumor’s low sensitivity.^[5]^

While RPLs are well-documented, cases involving extension into the thigh are exceedingly rare, with only a few reported in the literature. Such atypical presentations pose unique diagnostic and surgical challenges, requiring precise imaging to delineate tumor extent and meticulous surgical planning to navigate complex anatomical structures. Despite advancements in high-resolution computed tomography (CT) and magnetic resonance imaging, their role in guiding surgical interventions for these rare anatomical extensions remains underexplored.^[8]^

This report presents a rare case of RPL with thigh extension, emphasizing the diagnostic complexities, surgical considerations, and pathological findings associated with this atypical presentation. By contributing to the limited literature on this topic, we aim to enhance clinical understanding and provide insights into optimal management strategies for future cases.

## 2. Case presentation

A 45-year-old South Asian female with no known comorbidities presented to The Civil Hospital, Karachi, with progressive swelling in the left upper thigh. The swelling was first noticed 7 months prior as a small mass and gradually enlarged to involve the left inguinal region and flank. The patient also reported intermittent pain in the left proximal thigh, radiating toward the left abdomen over the past 2 years (Table 1).

**Table 1:** Summary of patient clinical events and management timeline.

Event	Timeframe
First noticed swelling	7 months ago
Pain onset	2 years ago
Referred to the surgeon	1 month ago
CT and biopsy performed	2 weeks ago
Surgery performed	Day 0
Discharged home	Day 5
First follow-up	1 month post-op
Second follow-up	6 months post-op (no recurrence)

CT = computed tomography.

She was referred by a general practitioner to the outpatient surgical clinic after a progressive increase in the swelling. The patient had a body mass index of 26.4, was a nonsmoker, nonalcoholic, and had no history of malignancy, metabolic disorders, or genetic syndromes. Her past surgical history was significant for a cesarean section performed 11 years ago.

On physical examination, a non-tender, firm, and diffuse swelling was palpated along the left flank, left inguinal region, and upper thigh. The mass was nonpulsatile, without overlying skin changes, signs of inflammation, or a cough impulse. The patient was afebrile, and her vital signs were stable.

### 2.1. Preoperative laboratory and imaging findings

#### 2.1.1. Laboratory results

Preoperative laboratory tests showed hemoglobin of 13.1 g/dL, total leukocyte count of 9.2 × 10⁹/L, and platelet count of 256 × 10⁹/L, all within normal limits (Table 2). Renal function markers urea (12 mg/dL) and creatinine (0.8 mg/dL) were normal. Liver function tests, including alkaline phosphatase (147 IU/L) and SGPT (19 IU/L), were also within normal ranges.

**Table 2:** Preoperative laboratory test results with corresponding normal reference ranges.

Test	Result	Normal range
Hemoglobin	13.1 g/dL	12–16 g/dL
Total leukocyte count	9.2 × 10^9^/L	4.0–10.0 × 10^9^/L
Platelets	256 × 10^9^/L	150–400 × 10^9^/L
Urea	12 mg/dL	7–20 mg/dL
Creatinine	0.8 mg/dL	0.6–1.2 mg/dL
Sodium (Na^+^)	142 mmol/L	135–145 mmol/L
Potassium (K^+^)	3.9 mmol/L	3.5–5.1 mmol/L
Chloride (Cl^−^)	104 mmol/L	96–106 mmol/L
Total bilirubin	0.3 mg/dL	0.1–1.2 mg/dL
Alkaline phosphatase	147 IU/L	44–147 IU/L
SGPT	19 IU/L	7–56 IU/L

SGPT = serum glutamic pyruvic transaminase.

#### 2.1.2. Imaging findings

**Figure 1.**
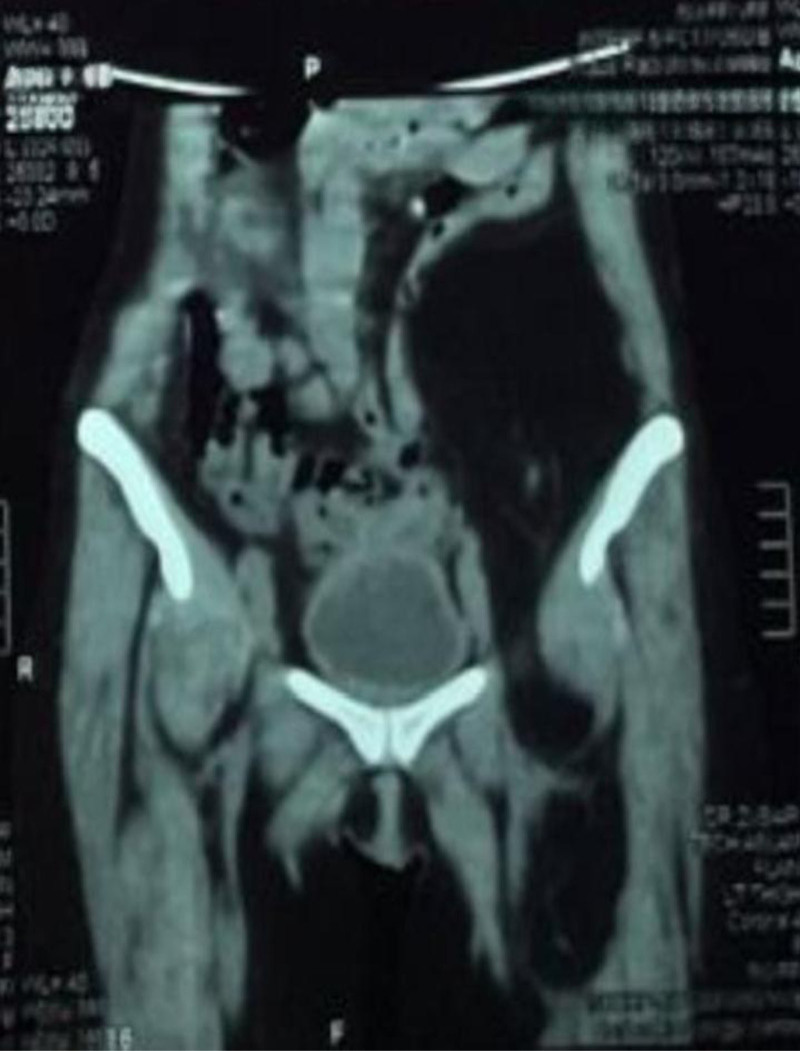
Imaging showing the lobulated, hypodense fat-attenuating lesion in the anterior compartment extending from the left retroperitoneal region into the left thigh.

**Figure 2.**
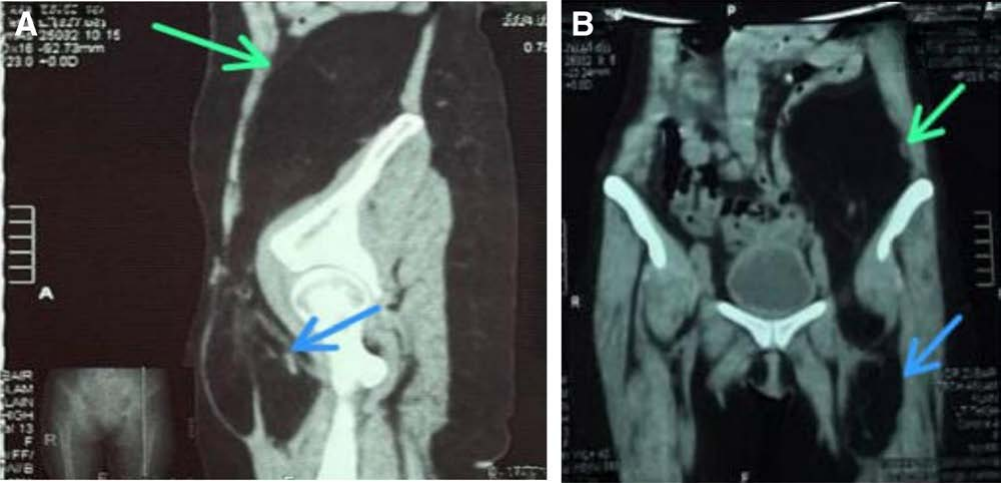
Retroperitoneal liposarcoma extending from the abdomen (green arrow) to the left thigh (blue arrow).

**Figure 3.**
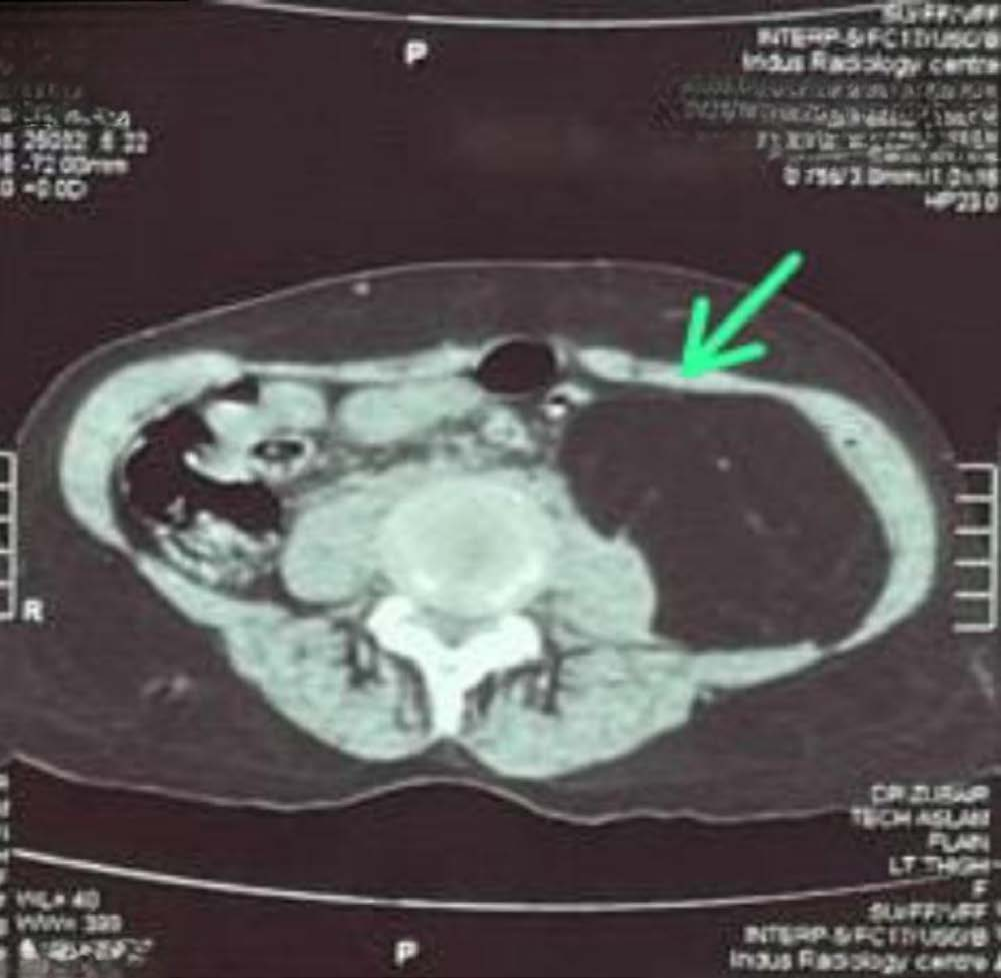
Liposarcoma in the retroperitoneum displacing other structures but with intact fat.

A contrast-enhanced CT scan of the left thigh and pelvis revealed:

- A large, lobulated, hypodense fat-attenuating lesion in the anterior compartment of the left proximal thigh, extending superiorly into the left pelvic and retroperitoneal region (Fig. 1).
- The lesion measured 11 × 9 cm in the axial plane, with a craniocaudal extent of 30.7 cm (Fig. 2).
- The mass displaced surrounding structures while maintaining intact fat planes, as shown in Figure 3.
- Multiple internal septations and areas of calcification were observed.
- No evidence of necrosis, vascular invasion, or aggressive features was noted.

### 2.2. Surgical approach and intraoperative findings

A combined laparotomy-thigh approach was chosen due to the tumor’s extensive craniocaudal length (30.7 cm) and the requirement for en bloc resection. The procedure lasted 4.5 hours and resulted in complete tumor excision with an estimated blood loss of 300 mL. No intraoperative neurovascular injury occurred. A postoperative seroma developed but resolved with drain retention for 7 days.

Preoperative radiation therapy was not pursued, as core biopsy confirmed a well-differentiated liposarcoma, which is known for its low radiosensitivity. In addition, concerns about radiation-induced fibrosis potentially complicating dissection planes supported the decision to proceed directly with surgical excision.

### 2.3. Differential diagnosis and histopathological confirmation

The differential diagnosis included:

- Retroperitoneal lipoma.
- Well-differentiated liposarcoma.
- Soft tissue sarcoma (other subtypes).
- Liposclerotic myxoma.
- Benign fibrous histiocytoma.
- Atypical lipomatous tumor.

Histopathological examination of the resected tumor confirmed well-differentiated adipocytes with scattered bizarre stromal cells.

- Immunohistochemistry showed p16-positive staining, confirming well-differentiated liposarcoma (grade 2).
- No dedifferentiation, necrosis, lymphovascular invasion, or pleomorphism was noted.
- Final diagnosis: well-differentiated liposarcoma.

### 2.4. Surgical and pathological findings

**Figure 4.**
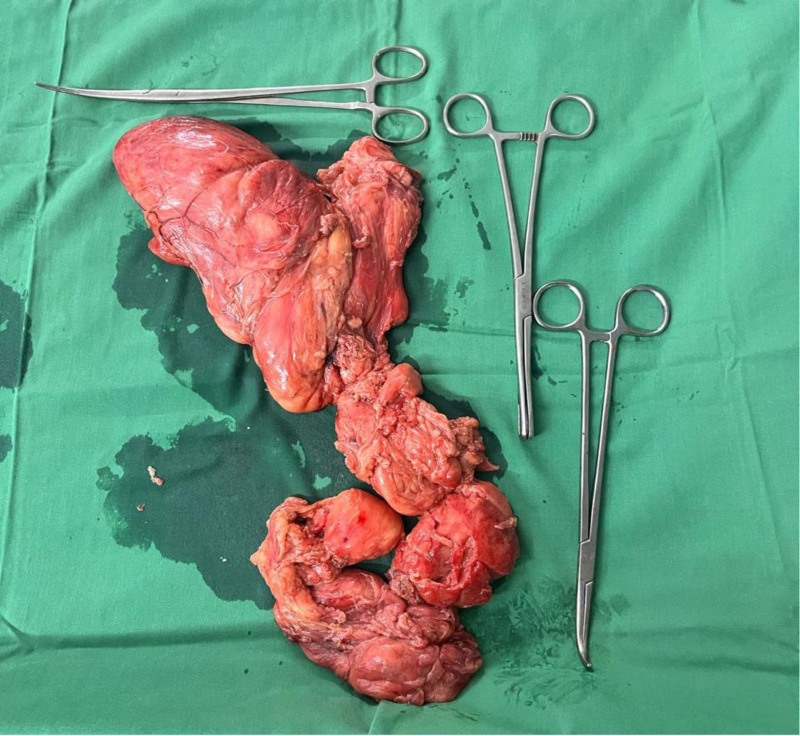
Intraoperative image showing the resected left-sided retroperitoneal lipomatous mass extending into the left thigh. The mass exhibits well-differentiated lipomatous features without evidence of capsule rupture.

The surgery was performed under general anesthesia with the patient in a supine position. A midline laparotomy was performed to access the retroperitoneal mass, revealing a 12 × 13 × 5 cm tumor extending along the left pelvic sidewall into the anterior thigh compartment. Due to its extensive craniocaudal involvement, a secondary vertical incision over the left anterior thigh was required for complete tumor mobilization and enucleation. The mass was carefully dissected from surrounding structures to preserve neurovascular integrity and avoid intraoperative complications. Intraoperative findings confirmed a well-differentiated lipomatous tumor with a fibrous capsule, without signs of necrosis or dedifferentiation. The resected specimen is shown in Figure 4, demonstrating the characteristic gross appearance of an RPL extending into the thigh.

A combined approach ensured complete tumor removal without capsule rupture. Drains were placed in the retroperitoneum and anterior thigh to prevent postoperative seroma formation. The abdominal fascia was closed with polydioxanone sutures, and the skin was closed with subcuticular Vicryl sutures.

#### 2.4.1. Perioperative prophylaxis included

- Intravenous cefazolin (1 g pre-op and 24-hour post-op); enoxaparin (40 mg subcutaneously for thromboprophylaxis); and multimodal analgesia (paracetamol + tramadol for 48 hours post-op).

The procedure was performed by a senior consultant surgical oncologist, assisted by a fellow in oncologic surgery and a senior resident.

### 2.5. *Postoperative recovery and follow-up*

#### 2.5.1. Postoperative laboratory findings. 

The postoperative lab findings were within normal limits. Hemoglobin was 10.2 g/dL, total leukocyte count was 10.3 × 10^9^/L, and platelet count was 235 × 10^9^/L. Overall, the lab results indicate no significant postoperative abnormalities. Histopathology confirmed no residual tumor, no dedifferentiation, and clear surgical margins.

At the 6-month follow-up, the patient:

- Reported no recurrence or new symptoms.
- Had no functional limitations.
- Follow-up imaging showed no residual or recurrent tumor.
- Scheduled for annual imaging surveillance.

The patient expressed relief at symptom resolution and reported improved quality of life. She resumed daily activities without restrictions and was satisfied with the surgical outcome.

## 3. Discussion

Retroperitoneal tumors (RPTs) are rare mesenchymal neoplasms that develop in the retroperitoneum, bordered posteriorly by the transversalis fascia and anteriorly by the posterior parietal peritoneum.^[9]^ These tumors originate from soft tissues such as fat, muscles, nerves, lymphatic structures, and blood vessels, with potential extension into intrapelvic organs (bladder, uterus, ovaries, and prostate) and retroperitoneal organs (kidneys, adrenal glands, and pancreas).^[10]^ While retroperitoneal soft tissue sarcomas typically remain confined, only 10% exhibit metastatic disease at presentation, with preferential hematogenous spread to the liver or lungs.^[11]^

RPTs can spread via 4 key mechanisms: hematogenous dissemination, lymphatic extension, perinephric bridging septa, and interfascial plane invasion.^[12]^ Among these, the iliopsoas compartment, which contains the psoas and iliacus muscles, serves as a crucial route for tumor progression due to its lack of distinct fascial boundaries, facilitating tumor extension into the pelvis and thighs.^[13]^ This case demonstrated an extensive craniocaudal tumor spread of 30.7 cm, an exceptionally rare presentation. RPLs commonly extend locally, but thigh invasion remains uncommon, with only a few cases reported. The tumor’s lobulated, fat-attenuating features, multiple internal septations, and calcifications further contributed to its distinct radiological appearance.

Preoperative contrast-enhanced CT played a critical role in assessing tumor size, extent, and anatomical involvement. The lesion measured 11 × 9 cm, with a craniocaudal spread of 30.7 cm, significantly influencing the surgical approach. In most RPT cases, imaging primarily focuses on tumor boundaries, vascular involvement, and adjacent organ relationships. While ultrasound is often used for initial soft tissue mass evaluation, CT and magnetic resonance imaging remain the gold standard for tumor characterization and preoperative planning.^[14]^

CT is particularly useful for identifying fat-containing tumors, such as well-differentiated liposarcomas, which typically appear as well-defined masses with thin septa. However, imaging features such as nodularity (suggesting dedifferentiation), thickened septations (>2 mm), and deep tumor location may indicate malignancy.^[15]^ In this case, internal septations and calcifications helped distinguish it from benign lipomatous tumors. Despite its advantages, CT has limitations, particularly in detecting microscopic invasion or small metastatic lesions, necessitating postoperative imaging surveillance.^[14]^

Surgical resection remains the gold standard for RPLs, as they demonstrate poor response to chemotherapy and radiotherapy.^[16]^ Given the high recurrence rates, achieving negative surgical margins is critical for long-term disease control.^[5]^ In this case, a combined laparotomy and anterior thigh approach was necessary due to the tumor’s extensive spread. The primary intraoperative challenges included preserving major neurovascular structures (femoral nerve and iliac vessels), controlling intraoperative bleeding, and ensuring en-bloc tumor removal without capsule rupture. In RPTs with iliopsoas involvement, the femoral nerve and adjacent vascular structures are at high risk.

Histopathological analysis confirmed a well-differentiated liposarcoma. While well-differentiated liposarcomas have a low metastatic potential, they carry a high risk of local recurrence.^[5]^ Core biopsy performed before resection confirmed well-differentiated histology, indicating low sensitivity to chemotherapy and radiotherapy. Postoperative histopathology demonstrated mouse double minute 2 homolog positivity without evidence of dedifferentiation, further supporting the decision to forego adjuvant therapy. This approach aligns with National Comprehensive Cancer Network guidelines, which recommend observation for margin-negative, well-differentiated liposarcomas.^[5,17]^ Although radiotherapy is sometimes considered for dedifferentiated or high-grade liposarcomas, its role in well-differentiated subtypes remains unclear, particularly when negative surgical margins are achieved.

The patient recovered uneventfully, with no postoperative complications. At 6-month follow-up, no recurrence was observed. Current recommendations suggest long-term follow-up.^[17]^ The decision to use annual CT monitoring aligns with these recommendations, ensuring early detection of recurrence and guiding further management.

## 4. Conclusion

This case underscores the importance of recognizing atypical tumor extensions in RPLs and highlights the crucial role of preoperative imaging in surgical decision-making. The unusual thigh extension posed significant diagnostic and therapeutic challenges, requiring a multidisciplinary approach to ensure complete tumor excision while preserving neurovascular function. Given the rarity of such extensive retroperitoneal spread, this report contributes to the limited literature on extraperitoneal liposarcomas and provides valuable insights into optimal surgical and diagnostic strategies.

## Author contributions

**Conceptualization:** Abdul Muqeet.

**Methodology:** Abdul Muqeet, Emad Uddin Sajid.

**Data curation:** Emad Uddin Sajid.

**Writing – original draft:** Emad Uddin Sajid, Mohammad Faisal Ibrahim, Anfal Atif, Muhammad Imran Siraj, Nour Fakih.

**Project administration:** Mohammad Faisal Ibrahim.

**Writing – review & editing:** Anfal Atif, Muhammad Imran Siraj, Nour Fakih.

**Formal analysis:** Absar Hyder.

**Visualization:** Absar Hyder.

**Supervision:** Nour Fakih.
